# Where are the SVHCs? 10 years consumer’s ‘right to know’ about substances of very high concern

**DOI:** 10.1186/s12302-017-0122-0

**Published:** 2017-07-20

**Authors:** Ursula Klaschka

**Affiliations:** University of Applied Sciences Ulm, Prittwitzstr. 10, 89075 Ulm, Germany

**Keywords:** Articles, Consumers’ ‘right to know’, REACH Regulation Art. 33(2), Substances of very high concern (SVHCs)

## Abstract

**Background:**

The European chemicals regulation REACH includes the legal duty for suppliers to inform consumers on request about the presence of substances of very high concern (SVHCs) in articles. Since this requirement has been in force now for 10 years, the intention of this study was to find out whether information on SVHCs is adequately communicated to the consumer today. Data on the presence of SVHCs in articles were collected as a prerequisite for the subsequent requests for a targeted choice of articles to examine the operability of the ‘right to know.’

**Results:**

Literature data show that SVHCs have been measured and described in a large variety of commodities. 32% of 334 information requests for articles which were suspected to contain SVHCs were answered by suppliers and a minor number of these answers were of good quality. Only two respondents indicated the presence of SVHCs in their articles. Suppliers are not legally obliged to respond to requests if their articles are free of SVHCs. Therefore, the absence of a response might be interpreted as an indication that SVHCs are present below 0.1% in the articles in question. However, there are certain doubts that only two out of 334 articles suspected contain SVHCs.

**Conclusions:**

The data question whether the ambitious aims of the SVHC regime can be achieved under the present conditions. Measures are proposed on how to improve implementation of the information requirement and to amend the legal criteria in the upcoming REACH revision.

## Background

The European chemicals regulation REACH 1907/2006/EC [1] introduced a new communication duty concerning substances of very high concern (SVHCs) in articles (Art. 33). SVHCs are chemicals which are carcinogenic, mutagenic, toxic for reproduction, or very critical for the environment because they are persistent, bioaccumulative and toxic (PBT), or very persistent and very bioaccumulative (vPvB) or which cause concern for other reasons such as endocrine disruptors ([1] Art. 57). They are listed in the so-called candidate list [2] which is updated twice a year (last update 12/01/2017 with a total of 173 entries) and which should include all relevant currently known SVHCs by 2020 [3]. According to Art. 33(2), consumers have the right to receive information from the suppliers of an article who is “any producer or importer of an article, distributor or other actor in the supply chain placing an article on the market” [1 Art. 3 (33)] about the presence of any SVHC. This applies for SVHCs that exceed a threshold of 0.1% (weight/weight) in an article in terms of REACH, in a subassembly of an article [4, 5] or in its packaging. Information shall be provided within a time period of 45 days upon receipt free of charge. This information obligation is a very important element in the REACH regulation with the ambitious goal to minimize adverse effects for the consumer and the environment by increasing transparency and awareness on SVHCs in consumer articles, by helping the interested consumers to make informed purchasing decisions and by ensuring a safe use of articles, and it supports the efforts by producers to substitute SVHCs by less dangerous chemicals.

The threshold of 0.1% (weight/weight) is based on a pragmatic decision like many other thresholds in legislation. A concentration limit was preferred to the limitation of release rates, which would have been much more difficult to estimate and which would depend on many article-specific parameters. Articles that contain more than 0.1% SVHCs do not necessarily lead to an exposure of consumers. This might only be the case if consumers get in direct contact with such an article, and if the SVHC has the potential to migrate out of the material. However, at the end of their product lives, articles containing SVHCs can contribute to an increased exposure of SVHCs in the environment depending on the waste disposal method leading potentially to an indirect exposure.

Some organizations published sample letters or online forms to support consumers with their requests (e.g., [6]). The German Federal Environment Agency has recently published a smartphone application ‘Scan4Chem’ [7] which facilitates the information request further. The smartphone application ToxFox by Friends of the Earth Germany included the question about SVHCs in their tool in addition to endocrine substances in 2016 [8].

Suppliers are not able to inform consumers about SVHCs if they do not receive the necessary information from the upstream users. Therefore, the implementation of the information duty according to Art. 33(2) depends crucially on an efficient information transfer in the supply chain as required in Art. 33(1) [9, 10]. The SVHC provisions in REACH motivate innovations. Several companies increased their efforts and substitute SVHCs in their articles. They prefer suppliers who renounce SVHCs, and several retailers demand supply of ‘SVHC-free’ articles. There are companies that manage to eliminate the use of SVHCs in all their articles. Some companies started cooperation with non-governmental organizations to make their products safer. These companies know more about their articles and the materials they are made of. They are better prepared for upcoming authorizations and restrictions and hence save future expenses and efforts. The promotion of these endeavors in the public raises the company’s reputation and contributes to a more trustful relationship with consumers. Therefore, phasing out SVHCs will result in competitive advantages for these companies [11, 12].

Various guidance documents exist which support suppliers to fulfill the information duty toward consumers [13–15]. They also receive online help [2, 16, 17] or can attend workshops at national or international level [11, 18].

What is the quality of the answers received from suppliers? Is the information that customers receive on their SVHC request helpful for them? Previous studies had shown many obstacles in the implementation of Art. 33(2) in practice [12, 19–21]. The objectives of this study were to analyze the functioning of this risk communication duty and to make recommendations for a potential revision of REACH provisions, e.g., in the upcoming REACH review of the scope of Art. 33 by 2019 [1 Art. 138 (8)]. As the online tool offered by the German Federal Environment Agency was used for the requests made in this study, also the performance of this tool was evaluated.

The first step of this investigation consisted of a compilation of data on the potential or verified presence of SVHCs in consumer articles, which allowed in the second step to make targeted information requests for such concrete articles in the retail market. The answers to the information requests were compared with the results of other studies and examined in the light of the knowledge about the amounts and frequency of these substances as described in the first step.

## Methods

Data on the presence of SVHCs in articles were searched in the literature considering national surveillance programs, non-governmental organization activities, research studies, and publications by the European Chemicals Bureau.

In the second step, 513 consumer articles were selected from the retail market in the area of Ulm and Stuttgart between August 29 and October 24, 2016. Article groups were preferred which had a certain probability to contain SVHCs as found out in step one. Many articles sold by well-known, large, and/or multinational companies were considered, because their quantitative impact on the total amount of SVHCs present in consumer articles might be large and their share of the supply offered to consumers will be larger compared to small companies. The barcode numbers printed on the labels of the articles were entered into the online tool offered by the German Federal Environment Agency [6]. Currently, the online tool is being reprogrammed and therefore not available. The smartphone app Scan4Chem [7] may be used instead. No requests were made by other means, such as by written letters via postal mail, by fax, by phone, or by direct face-to-face communication at the retailer of the articles. Requests were made preferentially for several articles of the same article category originating from different brands. This mimics the situation of a consumer who wants to buy a certain article without SVHCs and who does not want to wait again up to 45 days if the first article response declared the presence of SVHCs. Interested consumers would make several simultaneous information requests for comparable articles. The answers received by the suppliers or distributors were collected and analyzed and compared to results by previous studies.

## Results and discussion

### SVHCs in articles

The following sections assess which SVHCs, depending on their functions and chemical characteristics, are likely to be found in certain article categories and not in others. This allowed to select article categories presumed to contain SVHCs for the next tier of the investigation where information requests were sent to the article suppliers.

Table 1 shows a selection of SVHCs with examples of their potential presence in consumer articles. Data from the registration dossiers [22] indicate the annual amounts produced or imported into the European Union (Table 1, right column). It is important to note that most SVHCs are not only used in consumer articles, but also in other applications. Hence, only a fraction of these amounts is actually present in consumer articles. Manufacturers who use more than one ton SVHCs per year in articles (content exceeding 0.1%) must submit a notification of this use to ECHA (Table 1, column 1), unless exposure of humans or the environment can be excluded or unless the use is covered in the registration dossier for the substance [1 Art. 7 (2)]. Therefore, all notified substances in Table 1 are or will be used in applications with a certain exposure. Data in Table 1 are based on data provided by producers and importers in the REACH process for the European Union [23]. Up to October 2016, there were only 365 notifications for 39 candidate substances in articles [18], which is below the expected number of notifications. The question is whether other uses have been terminated and are no longer relevant, whether many notifications must still be expected, or whether other usages are covered in the registration dossiers. The low figure of notifications according to REACH Art. 7 ‘is likely to illustrate a low level of compliance’ [24].

**Table 1:** Examples of SVHCs in consumer articles and notifications by producers

Notified SVHC (number of notifications^1^) CAS No.	Examples of use in articles for consumers	Annual production volumes in tons per year^2^
Boric acid (15) 10043-35-3, 11113-50-1	Articles for decoration made from ceramic, articles from glass or ceramic intended for food contact, e.g., table ware, cups and plates, photo paper	100,000–1,000,000
Disodium tetraborate, anhydrous (10) 1303-96-4, 1330-43-4, 12179-04-3	Food contact materials, glass articles and ceramic articles, photo paper, waste ink absorbers	100,000–1,000,000
Lead monoxide (lead oxide) (7) 1317-36-8	Automotive batteries, accumulator, piezoceramic sensor	100,000–1,000,000
Pitch, coal tar, high temp. (3) 65996-93-2	Clay target	100,000–1,000,000
DEHP (132) 117-81-7	A large variety of articles, mainly plastic articles: flooring, artificial leather, toys, insulation material for cables and electric and electronic devices, packaging material	10,000–100,000 Annex XIV^3^ No. 4 Annex XVII^4^ No. 51
Diazene-1,2-dicarboxamide (C,C′-azodi(formamide)) (14) 123-77-3	Luggage and leather goods, plastics, rubber, wallpaper	10,000–100,000
1-Methyl-2-pyrrolidone (9) 872-50-4	Flexible PVC hose	10,000–100,000
HBCDD (35) 25637-99-4, 3194-55-6, 134237-50- 6, 134237- 51-7, 134237-52-8	Products with insulation materials	10,000–100,000 Annex XIV No. 3
Dibutyl phthalate DBP (20) 84-74-2	A very large variety of articles, mainly soft plastic articles: flooring, artificial leather, toys, insulation material for cables and electric and electronic devices, packaging material	1000–10,000 Annex XIV No. 6 Annex XVII No. 51
Bis(pentabromophenyl) ether (decabromodiphenyl ether, DecaBDE) (7) 1163-19-5	Non-woven fabrics	1000–10,000
Diboron trioxide (7) 1303-86-2	Articles from glass or ceramic for decorative purposes, or intended for food contact, generator and marine engine	1000–10,000
Lead sulfochromate yellow (C.I. Pigment Yellow 34) (4) 1344-37-2	Building material, fences, inflatable articles	1000–10,000
Lead chromate molybdate sulfate red (C.I. Pigment Red 104) (4) 12656-85-8	Building material, fences, inflatable articles, mat, signs, tarpaulin covers, tent	1000–10,000
Benzyl butyl phthalate (BBP) (3)85-68-7	Medical disposables, outdoor seating furniture, plastic packaging, power cord	1000–10,000 Annex XIV No. 5
Cadmium (1) 7440-43-9	Battery or battery pack	1000–10,000
Lead titanium zirconium oxide (9) 12626-81-2	Piezoceramic sensor, ultrasonic spray films in a humidifier	100–1000
2-(2H-benzotriazol-2-yl)-4,6-ditertpentylphenol (UV-328) (5) 25973-55-1	Labels, reflective signs, safety glass sheets and windshields	100–1000
Diisobutyl phthalate DIBP (19) 84-69-5	A very large variety of articles, mainly soft plastic articles: e.g., flooring, artificial leather, toys, insulation material for cables and electric and electronic devices, packaging material	1–10 Annex XIV No. 7
Alkanes, C10-13, chloro (short-chain chlorinated paraffins) (7) 85535-84-8	Electronic articles and accessories, lamps and microwave dishes, luggage and leather goods, plastic articles	Annex XVII No. 42
4-Nonylphenol, branched and linear, ethoxylated (5)	Safety glass sheets and windshields	Annex XVII No. 42
Lead chromate (2) 7758-97-6	Building material, fences, inflatable articles, mat, signs, tarpaulin covers, tent	Annex XIV No. 10 Annex XVII No. 28 Toxic to reproduction category 1
Aluminosilicate refractory Ceramic Fibers (21) (no CAS No)	Insulation articles	

^1^Number of notifications by producers as indicated in [23]

^2^Annual production volumes given are the amounts used in articles and non-articles according to the REACH registration dossiers [22]

^3^Annex XIV: list of substances subject to authorization [1]

^4^Annex XVII: restrictions on the manufacture, placing on the market and use of certain dangerous substances, preparations and articles [1]

While Table 1 column 2 contains the potential presence of the respective SVHCs in consumer articles, the following paragraph compiles information on the article-specific occurrence of SVHCs, such as governmental or non-governmental surveillance data, substance estimations in articles, or guidelines for environmentally friendly products.

Surveillance programs were initiated to control the presence of substances prohibited in articles [25] and to enforce the SVHC provisions relating to articles. Some data on these activities are published in the annual reports of the responsible national authorities. It must be emphasized that the surveillance programs by national as well as by non-governmental programs focus on about a dozen SVHCs, whereas the number of SVHCs amounts to currently 173. The small number of substances monitored is due to various reasons, such as the practicability of the analytical methods, the costs of expensive articles, the handling of very bulky or heavy articles, or the fact that some SVHCs undergo chemical reactions in the production process and might then stay below the trigger value in the final article. Furthermore, the surveillance of the same substances over several years allows to detect trends. The surveillance programs conducted by the enforcement authority of the German Federal State Baden-Württemberg focused on a few article groups where SVHCs or restricted substances [1 Annex XVII] might be expected. In 2013, the analytical measurements conducted in their surveillance programs revealed that SVHCs and restricted chemicals were present in many consumer articles, in some cases at rather high concentrations. Two-thirds of the articles under scrutiny contained SVHCs above the threshold of 0.1% and many answers by suppliers were wrong. 22 out of 84 articles contained more than 0.1% of the respective phthalates DEHP (up to 19%) and DIBP, whereas the producers had not indicated these SVHCs in their Art. 33(2) answers. The authorities analyzed up to 15 different phthalates, some of which are SVHCs (di-iso-heptyl phthalate (DIHP), bis-(2-methoxyethyl)phthalate (BMEP), *N*-pentyl-isopentylphthalate (PIPP), dipentyl phthalate (DPP), diisopentylphthalate (DIPP), dihexylphthalate (DHP)), some are SVHCs and listed in Annex XVII [1] (dibutyl phthalate (DBP), bis-(2-ethylhexyl)phthalate (DEHP), benzylbutylphthalate (BBP), di-iso-butylphthalate (DIBP)), and some of them are listed in Annex XVII and are no SVHCs up to now (dioctyl phthalate (DnOP), di-iso-nonylphthalate (DINP), di-iso-decylphthalate (DIDP)). Some phthalates that had previously been used as substitutes have meanwhile been regulated. This example of phthalates shows the problem, which surveillance organizations as well as producers have to face when provisions for a certain substance might change in the course of the years as the candidate list, Annex XVII, or other regulations are updated. Persistent organic pollutants such as short-chain chlorinated paraffins and HBCDD were also detected in various articles by the surveillance authorities of Baden-Württemberg. Cadmium was measured in articles with PVC material, jewelry, hard solder, and packaging. The biocide DMF was found in shoes and other leather articles. Lead (up to a concentration of 62%) and cadmium (up to a concentration of 0.18%) were measured in certain parts of electronic equipment. According to the data available in the annual reports between 2010 and 2014 [12, 26], the number of complaints remained at the same level during these years.

In 2011, the Environment Agency Austria tested phthalates and found di-iso-butylphthalate (DIBP) (36% w/w in plastic mats for children), di-iso-nonylphthalate (DINP)(43% w/w in massage balls), and di(ethylhexyl)terephthalate (up to 10% in inflatable outdoor children pool products) [27].

In 2016, the Swedish Chemicals Agency examined the presence of lead, cadmium, certain phthalates, short-chain chlorinated paraffins, and brominated flame retardants in a random check of 154 electrical and electronic articles. They detected prohibited substances in 38% of the articles which had to be withdrawn from the stores. Furthermore, six articles contained SVHCs above 0.1% [28].

In 2010, the Nordic council estimated in comprehensive case studies in cooperation with producers the realistic presence of SVHCs in the following articles: an upholstered sofa might contain hexabromocyclododecane (HBCDD) (5–10% w/w), formaldehyde, phthalates (DEHP, DBP, and BBP), chromium, azodyes, pigments, and organo-tin compounds (TBTO). A sports shoe might contain azodyes, dispersive dyes, formaldehyde in the textile parts, chromium VI, PCP, short-chain chlorinated paraffins in the leather parts, cadmium, lead, dimethylformamide, aromatic solvents, DEHP, DBP, and BBP in the plastic parts. A pliers might contain DEHP, DBP, and anthracene in the handles and chromium VI in the metal parts. A power distribution unit might contain DEHP in the PVC wire coatings and brominated flame retardants. A desktop computer might contain DBP (15% w/w in flexible PVC), DEHP (30–45% w/w in flexible PVC), BBP (30–45% w/w in flexible PVC), HBCDD (5–7% w/w in polystyrenes), short-chain chlorinated paraffins, and TBTO [29].

A group of European competent authorities provided practical advice for suppliers on the SVHC information duty and described the potential SVHC content of a selection of exemplary articles [13]. The following articles were identified which could contain SVHCs above the trigger value of 0.1%. A red plastic garden chair contained lead chromate molybdate sulfate red (C.I. Pigment Red 104). The handlebar grips of a bicycle contained DEHP and the seat covering contained dibutyl phthalate (DBP). A sofa covering consisting of textile contained HBCDD and the polyurethane cushion contained 0.2% tris(2-chloroethyl)phosphate. The PVC coating of a cable contained benzylbutylphthalate (BBP). A T-shirt with a print contained DEHP. A printed circuit board contained dibutyl phthalate (DBP), capacitors contained dibutyl phthalate (DBP), and a frying pan contained PFOA [13].

Substances of very high concern and restricted chemicals in articles were also monitored by non-governmental organizations. Azodyes, 3,3′-dimethoxybenzidine, 4-aminobenzidine, p-cresidine, and allergenic and carcinogenic dyes were detected in textiles [30, 31]. Heavy metals such as chromium III, chromium VI, tin, cadmium, nickel, lead, and antimony were measured in textiles [30, 31]. HBCDD was detected in textiles [31] and in packaging [19]. TBTO and various fluorinated compounds were found in textiles [31]. Phthalates were found in textiles [30, 31] and in various plastic articles [21]. PAHs were detected in textiles [31] and in children’s rubber boots [32]. Nonylphenol was detected in textiles [31].

Turnbull described the presence of short-chain chlorinated paraffins, HBCDD, TBTO, DBP (15%), DEHP, and BBP (total phthalate content 30–45%) in electronic equipment [33].

Criteria of voluntary product eco-labels such as the European eco-label [34], the German Blue Angel [35], or the Nordic Swan [36] often include requirements for hazardous substances. Most basic criteria for eco-labels for articles in terms of REACH comprise restrictions of SVHCs. The European eco-label is very strict and may not be awarded to SVHC-containing goods [34 Art. 6(6)]. Other eco-label criteria are a rich source for article types which might contain SVHCs (above or below the 0.1% trigger limit), unless they fulfill the ambitious eco-label standards. Just to name but a few examples of basic criteria in the German Blue Angel regime for articles: the criteria RAL-UZ 120 for resilient floor coverings rule out SVHCs as plasticizers or flame retardants, RAL-UZ 132 for thermal insulation material exclude phthalates and halogenated organic flame retardants, RAL-UZ 171 for office equipment with printing function exclude the intentional addition of SVHCs in various materials, or RAL-UZ 119 for mattresses contain regulations on tin-organic substances (such as TBTO), fluorinated hydrocarbons (such as PFCs), and other halogenated organic compounds like flame retardants.

The ‘Oeko-tex standard’ is a private certification system for textiles. Products that fulfill its criteria are marked by the ‘Oeko-tex standard’ label [37]. These criteria include also the analytical determination of harmful chemicals which may not surpass certain concentration thresholds in the specific fabric. Some of the substances which Oeko-tex monitor are SVHCs, such as dyestuffs and pigments classified as allergenic, carcinogenic, or banned for other reasons (two azodyes are SVHCs), extractable heavy metals (various lead and cadmium compounds are SVHCs), flame retardant substances (such as HBCDD), organic tin compounds (such as TBTO), perfluorinated compounds, phthalates (such as DEHP, DBP, BBP), polycyclic aromatic hydrocarbons (PAH) (such as anthracene), nonylphenol, OP, OPEO, and NPEO (which are also SVHCs). These criteria indicate which SVHCs might be present in textiles above or below the 0.1% trigger value.

Some SVHCs, such as plasticizers, are usually present in high percentages in materials, so that many articles containing phthalates fall under the information duty. On the other hand, other SVHCs are present far below 0.1% in an article, and their presence does not need to be communicated although their applications are very critical under environmental aspects. This is, for example, the case for perfluorinated compounds which are used in textile finishing and have a very long half-life.

### Implementation of Art. 33(2)

#### Targeted information requests

Data compiled above in “SVHCs in articles” were a basis for the second step of the investigation, which consisted of Art. 33(2) information inquiries for articles which have a certain probability to contain SVHCs above 0.1%, such as articles made of soft plastic materials, coated water proof or printed textiles, or electronic equipment. Specimens selected belonged to article groups which had also been investigated in the case studies by the Nordic council of ministries [29] or which had attracted attention in surveillance programs, e.g., by the authorities in Baden-Württemberg [12, 26] or by non-governmental organizations, e.g., [21]. The intention was to cover a large range of article sectors (Table 2).

**Table 2:** Information requests conducted for 513 articles

	Number of requests	Number of requests which were not sent out for technical reasons	Number of answers received (number of days passed after sending of request)
Adhesives	9	0	2 (Ø 5 days)
Bags	26	16	1 (Ø 1 day)
Building	33	5	7 (Ø 16, 9 days)
Electric articles	103	54	17 (Ø 5 days)
Leather articles	26	8	8 (Ø 13, 3 days)
Metal articles	35	9	5 (Ø 14, 4 days)
Paints	3	1	1 (Ø 13 days)
Plastic materials	67	24	18 (Ø 9, 4 days)
Shoes	19	7	6 (Ø 51, 5 days)
Sports equipment	22	11	4 (Ø 12, 3 days)
Textiles	56	15	12 (Ø 6, 1 day)
Toys	34	11	13 (Ø 6, 1 day)
Vehicles	27	3	9 (Ø 25, 6 days)
Other	52	15	7 (Ø 9, 4 days)
Total	513	179	110

The article sectors followed the classification as in [23] (Note that adhesives and paints were taken up, although they are mixtures and no articles in the sense of REACH, but their containers are articles in the sense of REACH)

Information requests for 513 articles were sent out. 179 requests were refused in the online form, either because the EAN was not valid, or the e-mail address of the supplier was not available or incorrect, or because an automated answer invited to send a letter by postal mail. A frequent problem was that there are various article identification numbers on the labels (GTIN, EAN, UPC, MPN, article number) and some manufacturers set up their own barcode system, so that it is not always easy or even possible to find the correct number that was accepted by the online tool. Numbers with 9, 11, 12, 13, and 14 digits were accepted. In some cases, producers assign one single article number to a group of articles, especially for commodities with fast-changing collections such as clothes or shoes. The online tool can forward the requests only for articles with a number registered in the GS1 system [38]. However, some answers arrived although the online tool had sent an error message. In one case, the internet search revealed that the error message (‘E-mail address of the supplier invalid’) received upon the request originated from the year 2008. The experiences show that the maintenance of the company contact data in the GS1 database is decisive for the success of the online tool.

Upon the 334 requests that should have arrived at the respective suppliers, 110 (33%) answers were received.

Only two producers indicated that SVHCs were present in their articles: a producer of electronic equipment sent a table showing that three cables, an earphone, and an envelope for a pocket calculator contained DEHP above 0.1% and a watch and a lithium battery contained 1,2 dimethoxyethane above this value. A producer of a child car seat indicated that this article contained DEHP above the threshold. Four respondents mentioned a concrete SVHC, but confirmed that the concentration stayed below 0.1% (azodyes in a leather purse and a sports jacket, BPA in a drinking bottle, chromium VI in a pair of leather pants, DEHP and PAH in LED light bulbs).

There may be various reasons why 224 suppliers did not send a response to the requests. Companies might assume that they need not answer, if their articles contain less than 0.1% of any SVHC. It is true that in this case, a supplier of an article is not legally required to respond. However, ECHA states very clearly on its homepage that ‘Companies are obliged to provide information if their articles contain SVHCs. They should also inform you if there are no SVHCs in their articles.’ And ECHA continues ‘If companies do not reply, you should approach your Member State’s enforcement authority for REACH’ [39]. Enforcement is entirely the responsibility of the individual Member States. Hence, in the present case, the German enforcement authorities could have been informed that 224 information requests were not answered.

Another reason why suppliers might not have replied to the Art. 33(2) requests lies in the definition of ‘consumer.’ As the author and two students had sent most of their information requests with their names and e-mail addresses of their academic institutions, some producers might have assumed that such an e-mail address indicated that the request was not sent from a private consumer, but that it was part of a study where they are not legally obliged to answer. There is no definition of ‘consumer’ in the REACH regulation. A general definition of ‘a consumer is a natural person, who is acting outside the scope of an economic activity (trade, business, craft, liberal profession)’ [40]. However, only one single producer asked why the author was interested in the information. After I answered that I made a study, he sent his response nevertheless. Suppliers who noticed that the inquirers were members of universities are legally required to provide the required information at the time of supply of an article according to Art. 33(1), which relates to information transfer for articles intended for professional use. No supplier provided such an information. Only one producer out of the 110 answers gave detailed information about the article use for the university building. The suppliers of the 224 articles, where no answers arrived, did not inquire about the purpose of the requests. So it is not clear whether the university address was the reason for not responding.

100 answers arrived in less than 45 days (48 answers in up to 2 days and 68 in less than 10 days) (Table 3). 10 answers arrived after the 45-day period. (Two suppliers of shoes had sent their answers for five pairs of shoes 61, respectively 62 days after the request). 7 answers arrived by postal mail, the others by e-mail. 97 answers were in German, one was written in hardly understandable broken German, seven answers were written partly in German and partly in English, and five were in English. 99 answers were written in easily understandable language, while 10 answers were judged to be very difficult to comprehend for average consumers, because they were very technical or contained abbreviations of regulations that were comprehensible only for experts in these legal fields.

**Table 3:** Results of Art. 33(2) information requests on SVHCs in a targeted choice of articles

513	Information requests were made
334	Information requests were sent out by the online form
110	Answers were received to the information request
102	Answers consisted of easily understandable formulations and were not too complicated and technical^1^
100	Answers arrived in time
97	Answers were of the same language as the request
68	Answers indicated that the respondent had understood the Art. 33 information duty^2^
48	Answers arrived within 2 days
45	Answers were of medium length (1–2 pages)
38	Answers mentioned packaging
25	Respondents mentioned their efforts concerning the environmental and sustainability management of their company
22	Answers mentioned the date of the candidate list
16	Respondents mentioned their efforts to substitute SVHCs by less dangerous substances
11	Answers mentioned subassemblies
11	Answers referred to eco-labels
4	Respondents gave support for a safe handling
2	Answers indicated an SVHC content above 0.1%

^1^Criterion was the understandability by average students who are not familiar with REACH

^2^Criterion was that the answer contained correct statements about the elements of REACH

The lengths of the answers ranged from one line to 24 pages. 56 answers were shorter than half a page (35 answers consisted of up to three sentences). 45 answers were one to two pages long, a length that should be suitable to comprise meaningful information for the consumers. 10 answers contained enclosures such as test reports. One manufacturer answered although their products were mixtures and no articles in the sense of REACH and they were only obliged to reply to the SVHC content of the packaging.

77 answers were received from producers, 14 from retailers, and 19 from importers. However, it was not always easy to find out what role the respondent has in the supply chain. Four answers did not inform about SVHCs, but referred to a different contact person or institution, and no statement with regard to the Art. 33(2) question was included. 102 of the answers came from Germany, six answers from other countries in the EU (four from the Netherlands, two from Austria), and two answers came from a contact person outside the European Union (from Switzerland).

27 respondents gave explanations about REACH as well as about Art. 33(2). 36 respondents mentioned general aspects of REACH, and 46 respondents explained the information duty according to Art. 33(2). 55 suppliers mentioned neither REACH in general, nor Art. 33(2). 42 answers contained wording which shows that the respondents are not very familiar with Art. 33(2). For example, “Article X is REACH compliant” is not a correct answer to the question whether an article contains SVHCs or not. The presence of an SVHC above 0.1% is allowed, as long as the substance is not subject to authorization or a restriction, but must be indicated on request.

Two respondents confounded the Art. 33(2) right to know with the registration requirement. Four respondents wrote erroneously that they would not have the obligation to inform about the presence of SVHCs, either because the respective article was already placed on the market before REACH came into force or because textiles would not be liable to the REACH regulation, which shows that these suppliers did not understand their legal Art. 33(2) duty.

16 respondents declare that their article does NOT contain ANY SVHC. It must be assumed that these suppliers meant that their articles did not contain SVHCs above the threshold of 0.1%. No one can exclude that a substance is present below the detection limit. Furthermore, SVHCs may be present below 0.1% in the article without information obligation. Therefore, in a narrower sense these 16 answers are not correct. The same is true for respondents who declared that SVHCs were not added (three respondents), or who state (three respondents) that SVHCs are not known to be present in their articles. Other wording like “does not contain in accordance to REACH” (nine respondents) was inappropriate for the same reason. 38 used the correct formulation “…. does not contain any SVHC above 0.1% (w/w).”

27 respondents did not mention the article under consideration (no product name or EAN number). 41 respondents used a general answer for all their articles. 35 respondents used general declarations: “.. The article is safe…..” four companies answered the requests for several of their articles in a combined mail. 38 respondents refer to the SVHC content in the article and its packaging. 14 respondents referred to the content of SVHCs also in subassemblies. 59 respondents indicated that they had the information about the SVHC content from own analytical measurements (10), from the supplier (12), from independent test laboratories (11), or from all the three sources (26).

22 answers mentioned the date of the candidate list which was valid at the time (10 respondents just mentioned the “current list”). Some respondents mentioned concrete dates of the candidate list used. General formulations do not allow to discern which update of the candidate list was used “current list of candidates,” “according to the present state,” “in the list… regularly updated,” “we follow updates of the REACH regulation and the candidate list regularly,” and “of course, we would inform you about any changes.”

16 respondents mentioned their efforts to substitute SVHCs by less dangerous substances. Four respondents gave support for a safe handling of the article, whereas five respondents draw attention to predictable application conditions. No respondent mentioned the authorization requirement for SVHCs which are listed in Annex XIV, and no supplier of building materials referred to the indication of SVHCs in the declaration of performance according to European Construction Products Regulation No. 305/2011 [41] for the respective article.

25 respondents mentioned their efforts concerning the environmental and sustainability management of their company, but none of them mentioned whether they had a certified Environmental Management EMAS or ISO 14000. 11 respondents referred to various labels as “Blue Angel” (1, a leather bag RAL-UZ 148), “Bluesign” and “Greenpeace Detox Commitment” (1) “Oeko-tex-standard-100” (4) “CADS” (former CATS—Cooperation avoiding toxic substances) (4), and seal by the German Technical and Scientific Association for Gas and Water DVGW (1). No respondent referred to the SIN (‘Substitute It Now!’) list [42], which is a globally used database of chemicals set up by the environmental organization ChemSec which compiles 912 (update February 2017) substances of concern based on the REACH criteria for SVHC substances.

Answers should fulfill the criteria which are legally defined according to Art. 33(2): the supplier “shall provide the consumer with sufficient information, … to allow safe use of the article including, as a minimum, the name of that substance.” However, it must be interpreted what “sufficient information” means, at the very least:

- Inform correctly about the SVHC content of the article.
- Contain information about safe use of the article.
- Arrive within 45 days of receipt.

In addition, the author considers the following criteria as appropriate for a satisfactory communication with consumers:

- Contain a precise description of the article in question, subassemblies, and packaging.
- Give a correct description of the Art. 33(2) information duty in the REACH regulation.
- Answer also in case no SVHC is present above 0.1% in the article/subassemblies/packaging.
- In case an SVHC is present, refer to the actual candidate list (date).
- Contain complete substance information [e.g., (eco-)toxicological effects, functioning in the article, concentration range].
- Contain information on whether the substance is subject to an authorization process from (date) onwards.
- Contain information about efforts of the manufacturer to substitute SVHCs by less dangerous substances.
- In the case of building materials, contain a reference to the declaration of performance.
- Contain information about safe disposal of the article.
- Are in the same language as the request.
- Are in a language that is easy to comprehend for average consumers and not too technical.
- Are about one page long.
- Arrive in substantially less than 45 days, e.g., 10 days.

None of the answers received fulfilled all these criteria (Table 3). Nevertheless, six suppliers took great care to answer the requests individually and explained very well the situation of SVHCs in their articles. In contrast, 19 answers, which were obviously not carefully made, gave reasons for doubt whether the answers were correct. This is especially true if it is known that an article of this kind often contains an SVHC (e.g., various phthalates in flexible plastic articles). Since market surveillance authorities in Germany found regularly that the answers of one-third of the companies requested for SVHC information were wrong, there are reasonable doubts whether all 224 articles where the suppliers did not send information on SVHCs did really not contain SVHCs above the threshold.

There may be many reasons why the answers to the information requests in the present study were not very satisfying:

- There are companies that might not take their legal duties to inform about the contents of SVHCs seriously. One reason for this could be that the information duty is not very familiar among consumers, so companies seldom receive any requests. In a survey among mainly highly motivated and well-informed participants conducted by the author in 2016, only 14.4% of 1066 interviewees indicated that they knew this right for information.
- Few enforcement authorities have the personnel and financial means for intensive enforcement efforts, so that the external pressure is rather low.
- Producers and importers might have to spend a lot of efforts to get exact information about the SVHC content of each component of their more or less complex articles from the suppliers in the supply chain, especially if the articles are imported from outside the EU. As the candidate list is prolonged twice a year, companies must safeguard that their suppliers always inform them about the content of SVHCs of the updated candidate list. In addition, chemical analysis of SVHCs may be challenging as testing methods differ with different materials and sometimes it is not easy or even feasible to determine concentrations around 0.1% in all materials. Furthermore, some companies might be reluctant to transfer information in the supply chain as they consider it as confidential business information.
- The online tool is not suitable for all articles. Many companies use their own barcode numbers which are not administered by GS1.
- Some companies might not have organized internally who is responsible for the response to information requests by consumers.

The Art. 33(2) information request could be more effective if suppliers are supported by a sample letter or an online tool [9]. There are various proposals for support to transmit information in the supply chain (see, e.g., SVHC-Communicator [43]), but a sample letter to be used for the consumer request has still been missing. Box 1 shows a proposal for a standard answer fulfilling the information duty according to Art. 33(2) REACH.

#### Box 1 Sample response letter to consumer requests according to Art. 33(2) REACH

Dear Madam/Sir,

thank you for your request from (*date*) and for your interest in our article (*article description and name*) (GTIN/EAN), model-/article-No. (*number*).

The objective of the European Chemical Regulation REACH (Registration, Evaluation, Authorization and Restriction of Chemicals, EC No. 1907/2006) is to ensure a high level of protection of human health and the environment. As a customer, you have the right to obtain information whether “substances of very high concern” are present in an article above 0.1% (weight by weight) (REACH right to know according to Art. 33(2) REACH Regulation No. 1907/2006). These substances are, for example, carcinogenic, mutagenic, toxic for reproduction, or very critical for the environment. The list of these substances is updated regularly and published by the European Chemicals agency ECHA (http://echa.europa.eu/chem_data/candidate_list_table_en.asp).

**Table:** 

*A) in case there are no SVHCs present above 0.1%:*	B*) in case there are SVHCs present above 0.1%:*
The article (*article description and name*) in question, as well as its subassemblies and its packaging, does not contain any SVHC mentioned in the candidate list from (*date*) above 0.1% (weight by weight).	The article (*article description and name*) in question/the subassembly of the article in question (*precise description*)/ the packaging (*precise description*) contains the following SVHC which is listed in the current candidate list (*date*) above 0.1% (weight by weight) (*if possible, actual concentrations in the article/subassembly/packaging*): A (*name of the substance*), (*IUPAC name, Cas-No., EG-No.*). This substance is of very high concern because … (*description of the effects on human health and the environment, e.g., carcinogenic, mutagenic, toxic for reproduction, very critical for the environment because it is persistent, toxic, and accumulate in organisms or which causes concern for other reasons such as endocrine disruption*). This substance is classified and labeled as (*classification and labeling according to the CLP Regulation*). Substance A is presently subject to an authorization process from (*date*) onwards/ is presently not subject to an authorization process. In the article of interest, substance A has the function of (*description*) and is also frequently present in *(other consumer articles)*. In our company, work is in progress to find less hazardous substitutes for this compound in the article of your interest. B *(Name of the substance*) … C *(Name of the substance*) … We recommend the following measures to minimize risk: *(Description of safety instructions).* For a safe disposal of the article, please *(description of recommendation concerning the disposal).* *(In the case of building materials*) You find further information about our article in the declaration of performance.

The article contains also the following hazardous substances (*names and classification and labelings)* which are not classified as substances of very high concern.

The article fulfills also the following European regulations *(e.g., legal provisions for electric and electronic articles, toys, building materials, etc.)*

(*Eventually*, d*escription of the company’s efforts concerning environmental management systems, eco-labels, or other programs.*)

Please let us know if you have further questions. We will be happy to help you.

Best regards

#### Comparison with previous investigations of the Art. 33(2) information duty

The present investigation is in agreement with previous studies which reported a low response rate and which found a low rate of positive findings of SVHCs in articles. In 2010, the European Environmental Bureau [21] sent information requests for 158 articles in five different European countries. 50% of the addressees did not answer and 22% of the responses were considered as adequate. The response rate in Germany received by Friends of the Earth was 62%, the second highest after the Netherlands with 81%. Only one-third of the answers received in their study were correct and arrived in time [21]. In a study conducted by BEUC and affiliated organizations in nine European countries for 675 articles, only one German company had announced that three tool kits contained DEHP [19]. Information requests were made in the framework of national market surveillance programs as mentioned in “SVHCs in articles.” In subsequent years, about one-fourth of the actual answers to Art. 33(2) requests were non-compliant, in many cases because an SVHC present in the article was not communicated [26].

#### Comparison of online tools with smartphone applications

An online tool like the one that is offered by the German Federal Environment Agency, which was used in the present study, could also be accessed on the homepage of Friends of the Earth Germany in the past. In total, 1550 customer requests had been sent in 2013 and 1070 in 2014 via these online tools.

In comparison, the smartphone application ToxFox by Friends of the Earth Germany [8], which had been launched in 2012, offers information about endocrine substances in cosmetic products. ToxFox was upgraded on October 20, 2016 and includes the possibility to send SVHC inquiries for articles since. The great achievement of this tool is that all suppliers’ responses are saved and build a continuously growing database. This allows customers to receive the answers to some of their requests without delay. This is also a simplification for suppliers as they have the option to enter the data on the SVHC content of their articles beforehand into the ToxFox database and subsequently receive less consumer inquiries. However, data in the database will need to be updated each time the producer amends the chemical composition of an article and after each amendment of the candidate list. The answers that were received on requests sent via ToxFox so far were as heterogeneous as the answers analyzed in the present study. For similar reasons as described above in “Targeted information requests”, they were unsatisfactory to a large extent. In addition, among the 1500 answers received up to March 2017, there were only two articles which contained SVHCs above 0.1% (a camera and an epilator containing borates). The experiences with ToxFox support the suspicion raised above, that consumers are not adequately provided with the information about the SVHC content in consumer articles.

The Swedish online tool Tjek kemien [44] which has been online now for 3 years was used for 75,000 consumer requests and 15,000 companies uploaded their SVHC data in the connected Danish database so far [18].

Scan4Chem [7], the smartphone application for REACH requests launched by the German Federal Environment Agency, does not contain a database for supplier responses like ToxFox and Tjek kemien. Nevertheless, it simplifies the process for consumers by automatically generating and sending the request. The supplier has to respond directly to the consumer. There are plans to also connect Scan4Chem to a database in the future on a European scale. So far, there are no quantitative results on the use of Scan4Chem available.

These experiences show that the support for consumers by these digital tools is growing, but still not perfect.

## Conclusions

### Compliance after 10 years ‘right to know’

The REACH information right for consumers about candidate list substances has been in force now for nearly 10 years and several studies have revealed since, that a large number of suppliers do not fulfill their obligations as foreseen. The data presented show that some companies take their responsibility for a trustful communication with the consumer seriously and have a very ambitious management system for minimizing dangerous substances in their production chains. On the other hand, the quality of answers received by the majority of suppliers is still insufficient. This indicates that the situation has apparently not improved satisfactorily. The aims of the REACH ‘right to know’ are ambitious, but apparently many suppliers are not aware of their communication duty and the concomitant advantages for them. There are also some doubts whether all suppliers’ answers are correct. The number of respondents who announce that their articles contain more than 0.1% of an SVHC is extremely low considering the targeted choice of articles in the present study, while publicly available data indicate that a lot of consumer articles today still contain SVHCs above the threshold of 0.1%. Without chemical analysis, it is not possible to decide whether the answers received are correct or not. Such checks are under the responsibility of the national enforcement authorities. Companies could be fined if they do not meet their information duties. In Germany, the fines foreseen for administrative offence are up to 50.000 Euros [45], but apparently such a sanction has not been imposed so far. Competent authorities call also for sanctions in cases where the company did not act with due diligence and relied on the information received by the supplier without making sufficient efforts to find out whether this information is correct [12]. Reasonable diligence would furthermore include the duty to consider information in the press, data published by consumer organizations, news about article recalls due to SVHCs, or knowledge about the expected presence of SVHCs in a certain article group or material. Due diligence can also include analytical testing commissioned by the company. Companies that provided wrong information about the SVHC content in their articles are required by law to pay for costs resulting from analytical surveillance tests by the enforcement authorities [12].

‘Restrictions are after the fact’ [18]. This means in the case of SVHCs that restrictions are based on the identification of real unacceptable risks. As long as SVHCs are used in articles, exposures and risks are real. ‘Safety improved, but consumers are not yet safe’ [18]. Therefore, the violation of SVHC provisions should not be regarded as trivial offence and must be prosecuted. A general absence of legal consequences in case of non-compliance with the information duty is the wrong signal for companies who take their responsibility seriously, phase out SVHCs in their articles, and respond to all consumer requests. ECHA’s position is that Art. 33(2) is an important right of consumers and the fact that suppliers do not reply may be a breach of this provision. ECHA encourages the consumers to contact the responsible enforcement authorities if suppliers still do not send an answer upon a renewed request.

### Recommendations

#### Future revisions

The objective of the consumer’s ‘right to know’ is threefold: increase awareness on SVHCs in articles, support safe use of articles, and provide market incentives for substituting SVHCs. These aims are not fulfilled if the consumers do not seize the opportunity and request information, if the information is not understandable and workable for the average informed consumer, or if the respondents do not answer correctly or not at all. Practical experiences as presented here can contribute to the elaboration of feasible improvements in the course of the upcoming reviews of the REACH Regulation in the years to come (in 2017, second REACH review [1 Art. 117 (4)], 7th Environment Action Programme and the development of a non-toxic environment by 2018 [46], and assessment ‘whether or not to extend the scope of Art. 33 to cover other dangerous substances’ [1 Art. 138 (8)] by June 2019).

#### Improvements of effectiveness and effectivity of the current provisions

Optimized cooperation between ECHA and suppliers as well as education campaigns for industry and trade actors could support them on how to carry out their legal duties. Information flow in the supply chain can be simplified by a standardized format for communication on SVHCs in articles [9, 43]. If information requests are facilitated in all EU countries, e.g., by easy-to-use smartphone applications like ToxFox or Scan4Chem, the number of information requests by consumers could be increased, leading to an increased awareness of consumers as well as of suppliers. An official sample letter, such as the one proposed in Box 1, could facilitate the answers to the consumer requests for suppliers and could render the answers better understandable for consumers. However, an exclusive focus on SVHCs might distract consumers from other dangerous substances in everyday products. For example, if SVHCs are absent in the packaging of personal care products, this might lead to a false sense of safety as the personal care product itself in the container may contain heavy metals, potential sensitizers, or endocrine disruptors. The information duty should therefore be extended to mixtures (like personal care products) and it should not only include SVHCs, but also address substances that were identified to be of risk in other regulations (see also [47]). A full declaration of ingredients in articles could better cope with a growing candidate list and increasing information about other hazardous substances. The SVHC information does not give the consumer any information either about the chemical exposure by the respective article or about the aggregated exposure from various sources. This aspect should also receive more consideration in future. Improvements are needed in the suppliers’ responses to support safe handling of articles by consumers. In addition, clear criteria are needed for substituting substances which ascertain that the new ingredients are proven to be less dangerous than the substituted substance according to the present assessment criteria. The SINimilarity tool is a first help to avoid substitution by substances of similar hazard [42].

Effective surveillance and enforcement mechanisms by national enforcement authorities could lead to a fair situation which does not discriminate against companies that fulfill their duties well. In addition, more surveillance activities by authorities are needed to find out whether the information on the SVHC contents in the suppliers’ responses is correct or not. Fines or other enforcing measures that are already foreseen in legislation as described above should be applied to increase compliance. If consumers make informed purchasing choices and prefer SVHC-free articles, companies could profit from the competitive advantage using the information duty and the efforts to substitute SVHCs for a transparent and trustworthy communication with consumers.

So far, the current REACH information requirements for the supply chain are also applicable to articles produced in non-EU countries, but the enforcement is difficult. The Nordic Council estimated that large amounts of SVHCs are imported into the European consumer market, for example 900 tons per year of one SVHC with the import of shoes [29]. The application of SVHC regulations and especially the authorization requirement to imported articles is in line with WTO law as shown in previous studies [48–50]. Extension of the authorization procedure to imported articles is not a violation to WTO rules against protectionism. This would also reduce a serious market disadvantage of European manufacturers and would generate a level playing field for all economic actors [49, 50].

#### Further proposals

Alternative or supplementary information tools on SVHC contents of articles should be considered. This could be the labeling of all contained SVHCs or even the full declaration of hazardous ingredients of an article on a package leaflet. Such a list could also contain information about potential risks and recommendations for safe use. Another option would be to make information on SVHCs in articles available in a European public open access database, fed and updated regularly with SVHC information by the responsible suppliers. As long as the consumers cannot retrieve the SVHC information by such alternative means, the maximum time limit for the answer should be reduced from the given time of 45–10 days. The experiences in the present study show that the majority of suppliers sent their answers already today in less than 10 days. Up to now, it is unclear whether missing answers should be interpreted as ‘the article contains less than 0.1% of any SVHC’ or whether the information request was ignored in the company. Therefore, the consumer should receive a confirmation of receipt of a request and suppliers should be liable to answer consumer requests in all cases.

### It is worth to strengthen the ‘right to know’

REACH has established several elements that should lead to the gradual substitution of SVHCs: the information communication in the supply chain, the restrictions, the authorization requirement, and the consumer’s ‘right to know.’ The results suggest that the consumer’s ‘right to know’ in the present form is not very effective. More efforts are needed by all interested parties, i.e., regulators, manufacturers, importers, retailers, and consumers, to improve the implementation of this important information duty.

## References

[1] Regulation (EC) No 1907/2006 concerning the registration, evaluation, authorisation and restriction of chemicals (REACH). http://eur-lex.europa.eu/legal-content/en/TXT/?uri=CELEX:32006R1907. Accessed 8 Feb 2017

[2] https://echa.europa.eu/candidate-list-table. Accessed 8 Feb 2017

[3] https://echa.europa.eu/…/svhc_roadmap_implementation_plan_e. Accessed 8 Feb 2017

[4] European Court judgement. ….. Regulation (EC) No 1907/2006—articles 7(2) and 33—substances of very high concern present in articles—duties to notify and provide information—calculation of threshold of 0.1% weight by weight. Case 106/14 (2015). http://curia.europa.eu/juris/liste.jsf?&num=C-106/14#. Accessed 27 Jun 2017

[5] ECHA (2017) Guidance on requirements for substances in articles. https://echa.europa.eu/documents/10162/23036412/articles_en.pdf. Accessed 29 Jun 2017

[6] https://www.umweltbundesamt.de/en/topics/chemicals/reach-what-is-it/reach-for-consumers. Accessed 8 Feb 2017

[7] https://play.google.com/store/apps/details?id=de.uba.scan4chem, and https://itunes.apple.com/de/app/scan4chem/id1205416098. Accessed 20 Mar 2017

[8] https://www.bund.net/themen/chemie/toxfox/. Accessed 15 Feb 2017

[9] Reihlen A, Jepsen D, Wirth O, Bunke D (2015) Feasibility study “Supply chain communication on SVHC in Articles”. UBA Texte 102/2015. https://www.umweltbundesamt.de/sites/default/files/medien/378/publikationen/texte_102_2015_feasibility_study_supply_chain_communication_on_svhc_in_articles.pdf. Accessed 29 Jun 2017

[10] Reihlen A, Jepsen D, Bunke D (2015) Guidance on communication on substances in articles. UBA-Texte 103/2015. http://www.umweltbundesamt.de/publikationen/guidance-on-communication-on-substances-in-articles. Accessed 8 Feb 2017

[11] Oekopol (2013) SVHC-Stoffe in Erzeugnissen REACH-Pflichten vor dem Hintergrund weiterer regulatorischer Aktivitäten und Hilfen zu ihrer Umsetzung REACH in der Praxis III, Conference proceedings No 6, Berlin (in German). https://www.umweltbundesamt.de/en/node/28869. Accessed 8 Feb 2017

[12] Wursthorn S, Adebahr W (2013). Erfahrungen beim Vollzug der Informationsverpflichtungen nach Artikel 33 der REACH Verordnung (in German). StoffR.

[13] Belgian, German, Danish, French, Norwegian and Swedish chemicals agencies (2013) Guidance for suppliers of articles. The REACH duties to inform about candidate list substances. ISBN 978-91-7932-066-X. https://www.kemi.se/en/global/broschyrer/gui. Accessed 8 Feb 2017

[14] Führ M (2011). Praxishandbuch REACH (in German).

[15] BDI (2013) Stoffrecht. Hilfen zur Umsetzung der REACH- und CLP-Verordnung (in German). http://www.reach-compliance.ch/downloads/REACH_Brosch_201306.pdf. Accessed 8 Feb 2017

[16] http://www.reach-clp-biozid-helpdesk.de and http://svhc-in-articles-communication.de/index.php?id=home. Accessed 8 Feb 2017

[17] http://www.reach-info.de/chemikalien_in_erzeugnissen.htm. Accessed 8 Feb 2017

[18] Conference proceedings 2016. http://www.bfr.bund.de/de/uebersicht_der_praesentationen_zum_reach_kongress_2016__verbraucherschutz_unter_reach__am_5__oktober_2016-199135.html and http://www.bfr.bund.de/de/uebersicht_der_praesentationen_zum_reach_kongress_2016__verbraucherschutz_unter_reach__am_6__oktober_2016-199136.html. Accessed 21 Mar 2017

[19] BEUC (2011) How much are we told. http://www.beuc.eu/publications/2011-09794-01-e.pdf. Accessed 8 Feb 2017

[20] Chemical Watch (2010) Retailers in the dark over REACH right to know. https://chemicalwatch.com/3703/retailers-in-the-dark-over-reach-right-to-know. Accessed 8 Feb 2017

[21] EEB (2010) The fight to know? Substances of very high concern and the citizens. Right to know under reach. http://www.eeb.org/EEB/?LinkServID=8BBC1DF8-C9C7-8B93-CA5F42033F11A3AD. Accessed 8 Feb 2017

[22] https://www.echa.europa.eu/web/guest/information-on-chemicals/registered-substances. Accessed 8 Feb 2017

[23] ECHA (2016) Data on candidate list substances in articles. https://echa.europa.eu/documents/10162/13642/data_candidate_list_substances_in_articles_en.pdf. Accessed 8 Feb 2017

[24] https://echa.europa.eu/documents/10162/13634/operation_reach_clp_2016_en.pdf. ECHA-16-R-08-EN. ISBN: 978-92-9495-026-0. Accessed 8 Feb 2017

[25] https://echa.europa.eu/about-us/who-we-are/enforcement-forum/forum-enforcement-projects. Accessed 8 Feb 2017

[26] https://um.baden-wuerttemberg.de/de/service/publikationen. Accessed 9 Feb 2017

[27] Verein für Konsumenteninformation (VKI) (2001). Schadstoff - Informationen nur auf Anfrage (in German). Konsument.

[28] KEMI (2016) Tillsyn av elektriska lågprisprodukter (in Swedish). https://www.kemi.se/…/tillsyns…/tillsyn-11-16-tillsyn-av-elektrisk. Accessed 8 Feb 2017

[29] Nordic Council of Ministers (ed) (2010) REACH trigger for information on substances of very high concern (SVHC)—an assessment of the 0.1% limit in articles, TemaNord 514 Kopenhagen. ISBN 978-92-893-1998-0. http://www.norden.org/publications. Accessed 8 Feb 2017

[30] PROSAFE (2013) Joint market surveillance action co-funded by the European Union. Final technical report. Chemicals in clothing. Agreement No: 2013 82 01, D11.2CL. 2016. http://www.prosafe.org/images/Documents/JA2013/JA2013_D11.2CL_Technical_Report-chemicals_in_clothing.pdf. Accessed 8 Feb 2017

[31] BEUC (2016) Protecting consumers from hazardous chemicals in textiles. ANEC/BEUC contribution to the European Commission public consultation on possible restriction of hazardous substances (CMR 1A and 1B) in textile articles for consumer use. BEUC-X-2016-020–02/03/2016

[32] Oeko-test Test report 2011. http://www.oekotest.de. Accessed 8 February 2017

[33] Turnbull A (2008) REACH requirements for component suppliers and equipment manufacturers. ENVIRON http://www.BOMcheck.net. Accessed 8 Feb 2017

[34] Regulation (EC) No 66/2010 on the EU Ecolabel. http://eur-lex.europa.eu/legal-content/EN/TXT/?uri=celex%3A32010R0066. Accessed 27 Jun 2017

[35] https://www.blauer-engel.de. Accessed 8 Feb 2017

[36] http://www.nordic-ecolabel.org/criteria/product-groups. Accessed 8 Feb 2017

[37] http://www.oeko-tex.com. Accessed 8 Feb 2017

[38] https://www.gepir.de. Accessed 21 Mar 2017

[39] https://echa.europa.eu/chemicals-in-our-life/how-can-i-use-chemicals-safely/use-your-right-to-ask. Accessed 8 Feb 2017

[40] Mańko R (2013) The notion of ‘consumer’ in EU law—European Parliament—Europa.eu 130477REV1. Library of the European Parliament. 06/05/2013

[41] European Construction Products Regulation No 305/2011 of 9 March 2011 laying down harmonised conditions for the marketing of construction products and repealing Council Directive 89/106/EEC. http://eur-lex.europa.eu/legal-content/de/TXT/?uri=CELEX%3A32011R0305. Accessed 8 Feb 2017

[42] http://chemsec.org/business-tool/sin-list. Accessed 27 Jun 2017

[43] SVHC-Communicator http://svhc-in-articlescommunication.de. Accessed 28 Jun 2017

[44] http://tjekkemien.dk. Accessed 8 Feb 2017

[45] http://www.reach-info.de/chemikalien-sanktionsverordnung.htm (in German). Accessed 8 Feb 2017

[46] http://ec.europa.eu/environment/action-programme. Accessed 8 Feb 2017

[47] Kalberlah F, Augustin R, Bunke D, Schwarz M, Oppl R (2011) Karzinogene, mutagene, reproduktionstoxische (CMR) und andere problematische Stoffe in Produkten. Identifikation relevanter Stoffe und Erzeugnisse, Überprüfung durch Messungen, Regelungsbedarf im Chemikalienrecht (in German). Umweltbundesamt, Texte 18/2011. http://www.reach-info.de/chemikalien_in_erzeugnissen.htm. Accessed 8 Feb 2017

[48] Lüskow H, Heitmann K, von Bockum M (2010) Analysis of the realization of obligations from article 7 and 33 under REACH for imported articles. http://www.umweltbundesamt.de/importierte-erzeugnisse. Accessed 27 Jun 2017

[49] Schenten J, Führ M (2016). SVHC in imported articles: REACH authorisation requirement justified under WTO rules. Environ Sci Eur.

[50] Führ M, Schenten J, Hermann A, Bunke D (2015) Enhancement of the REACH requirements for (imported) articles. Options for improvement of the chemicals regulation Umweltbundesamt, Texte 41/2015. http://www.umweltbundesamt.de/publikationen/enhancement-of-the-reach-requirements-for-imported. Accessed 27 Jun 2017

